# A Review of Radio Frequency Identification Sensing Systems for Structural Health Monitoring

**DOI:** 10.3390/ma15217851

**Published:** 2022-11-07

**Authors:** Muchao Zhang, Zhaoting Liu, Chuan Shen, Jianbo Wu, Aobo Zhao

**Affiliations:** 1 School of Mechanical Engineering, Sichuan University, Chengdu 610065, China; 2 Nuclear Advanced Manufacturing Research Centre, iHub, Infinity Park Way, Derbyshire DE24 9FU, UK; 3 Seeneuro Ltd., 4th Floor, Building 4, No. 85 Keji Avenue, Yuhang Street, Hangzhou 310000, China

**Keywords:** radio frequency identification, sensor tags, structural health monitoring, battery-less, applications, structural health prognostics

## Abstract

Structural health monitoring (SHM) plays a critical role in ensuring the safety of large-scale structures during their operational lifespan, such as pipelines, railways and buildings. In the last few years, radio frequency identification (RFID) combined with sensors has attracted increasing interest in SHM for the advantages of being low cost, passive and maintenance-free. Numerous scientific papers have demonstrated the great potential of RFID sensing technology in SHM, e.g., RFID vibration and crack sensing systems. Although considerable progress has been made in RFID-based SHM, there are still numerous scientific challenges to be addressed, for example, multi-parameters detection and the low sampling rate of RFID sensing systems. This paper aims to promote the application of SHM based on RFID from laboratory testing or modelling to large-scale realistic structures. First, based on the analysis of the fundamentals of the RFID sensing system, various topologies that transform RFID into passive wireless sensors are analyzed with their working mechanism and novel applications in SHM. Then, the technical challenges and solutions are summarized based on the in-depth analysis. Lastly, future directions about printable flexible sensor tags and structural health prognostics are suggested. The detailed discussion will be instructive to promote the application of RFID in SHM.

## 1. Introduction

Structural health monitoring (SHM) acts a pivotal part in detecting, localizing, and assessing damage to large-scale structures (e.g., wind turbines, pipelines, and railways) at the early stage, which is a reliable, effective and economical monitoring method to ensure structural safety [1]. For example, the long-term alternating load generated by wheelsets will cause deformation, cracks, peeling and other damages to rails, which will reduce the stability and the safety of the train [2]. Pipeline cracks and corrosion can occur from the electrochemical and stress effects in service [3]. Mechanical failures of wind turbines require extremely high operation and maintenance costs. Therefore, SHM of large-scale structures, which aims at structural integrity and faults diagnosis, is critically important.

SHM based on wireless sensor networks (WSN) is an attractive option compared to the traditional wired system. Previous SHM adopted cables or wires as the basic tool to form a data acquisition system. This traditional signal transmission method not only has complex wiring but also increases the load of the monitored structures. In addition, the time and effort expended on the maintenance of cables and wires in the later period are also considerable. By removing wiring connections, WSN has more advantages in respect of installation. WSN consists of numbers of distributed sensor nodes that interact with the physical environment, such as humidity, temperature and pressure. These nodes not only have sensing ability but also have embedded processors and wireless communication elements. Therefore, various SHM designs based on WSN have been developed. Alves et al. [4] proposed the wind turbine SHM system called Delphos, which uses wireless sensor nodes composed of accelerometers to obtain blade natural frequency information to predict wind turbine damage. An SHM system was developed for a railway station, which used acceleration, stress, wind load and temperature sensors to obtain health data for the steel structures for the whole life cycle [5]. The WSN-based SHM system makes the large-scale wireless real-time monitoring of structures a reality. However, these nodes require batteries for long-term monitoring, which will increase the cost of large-scale applications for the maintenance and replacement of batteries. This undoubtedly promotes the case for using passive sensor nodes in the field of SHM.

Consequently, passive nodes based on radio frequency identification (RFID) are under investigation. An RFID system uses tags attached to the objects (to be identified) and a two-way radio transmitter-receiver (reader). Tags are powered by the energy from the interrogation radio waves of the reader. RFID tags have the advantages of simple circuit structure, low cost, being wireless, and having no battery. Unfortunately, RFID has a poor sensing capacity. WSN can sense environmental changes but requires batteries. Hence, RFID and WSN can be combined to exploit their advantages to form passive RFID sensor tags for SHM, such as antenna-based RFID sensor tags, digital integrated RFID sensor tags, etc.

**Figure 1:**
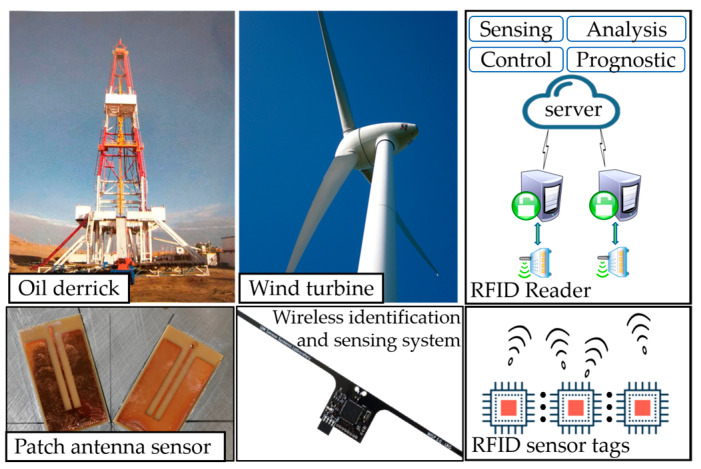
RFID sensor tags for SHM systems.

Recent works have demonstrated the potential of various RFID-based sensing techniques for SHM systems. Taking the RFID antenna as a sensor, it can detect cracks [6,7,8,9,10], corrosion [10,11,12,13], or stress [14,15,16,17,18,19] in structures. These defects can cause changes in the RFID signal, such as radar cross section (RCS) [20,21], received signal strength indicator (RSSI) [17,22], phase [10,23,24], S-parameters [25,26], and turn on power [9,15]. As a means of communication and energy harvesting, wireless sensor nodes formed by RFID combined with low-power sensing technology are also suitable for SHM applications. And, chipless RFID sensors have also attracted the attention of researchers due to their simple structure and ultra-low cost. Figure 1 shows examples of RFID sensor tags for SHM.

Several review papers [27,28] have studied the application of RFID for SHM. Nevertheless, previous work mainly focused on antenna-based tags for SHM and analyzed the communication and sensing issues. In addition to communication and sensing problems, there are still numerous scientific challenges to be addressed before the large-scale application of RFID-based SHM systems, for example, multi-parameters detection for robustness monitoring, the low sampling rate of RFID sensing systems and antenna design. After several years of development (2017–2022), the technical challenges faced in the past may have been solved or new solutions have emerged, such as strain direction or omnidirectional strain detection solution [18,25], and novel corrosion prediction methods based on chipless RFID [29]. To facilitate its application from simple laboratory stage modelling and testing to large-scale real-world structures, this paper presents a systematic review of RFID-based SHM systems. Current problems are analyzed and potential solutions are discussed. To this end, the remainder of this paper is organized as follows: Section 2 presents the method of the literature review. The fundamentals of RFID sensing systems are introduced in Section 3. Section 4 analyses designs and applications of RFID sensor tags in SHM according to their working mechanism. Section 5 discusses the technical challenges, solutions or future trends about the application of RFID sensing systems in SHM. Finally, the paper is concluded in Section 6.

## 2. Method

Articles were searched on the *web of science*. To obtain more comprehensive results, we collected articles with topics including radio frequency identification or RFID, as well as sensing or sensor(s). After removing irrelevant papers such as object management, more than 210 documents related to structural health monitoring were selected. The detailed information on retrieval and selection is shown in Table 1. This study finally selected these articles related to strain or stress, crack or corrosion, and vibration in SHM. The studies were divided into four topologies according to their working principle, i.e., COTS, antenna-based, digitally integrated and chipless RFID sensor tags. The following qualitative and quantitative analysis is based on but not limited to these articles.

## 3. Fundamentals of RFID Sensing System

RFID is a passive wireless identification technology that was used, among other things, during World War II to identify whether planes belonged to “friends or foes”. Unlike the bar code and the QR code, RFID does not require direct contact and line of sight. According to the frequency band used, RFID can be divided into low frequency (LF), high frequency (HF), and ultra-high frequency (UHF), as shown in Table 2. From LF to UHF, the data rate between a tag and a reader increases, but the performance near liquids and metals decreases.

**Figure 2:**
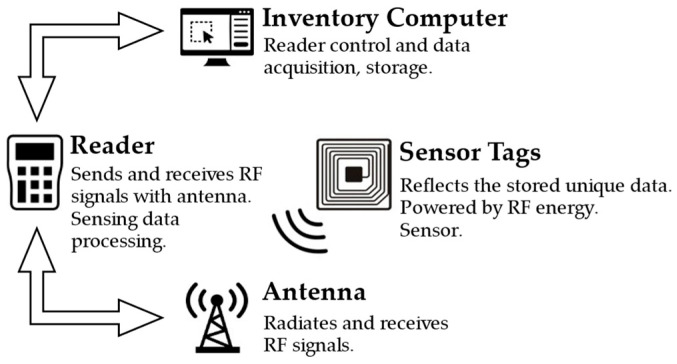
Basic RFID sensing system.

Once the sensing function is introduced, the RFID system will change into an RFID sensing system, which can be used in SHM. A typical RFID sensing system is depicted in Figure 2. The basic components of the system include the application software in the inventory computer, the reader, antennas and sensor tags. The reader along with the application software is the control center of an RFID sensing system. The reader is normally composed of the radio frequency (RF) signal transceiver module, the baseband signal processing module, the control module, and the interface module. A reader is a bi-directional radio transmitter-receiver that both modulates and demodulates radio waves. The reader can contain multiple antenna ports, and the number of antennas depends on the coverage area.

**Figure 3:**
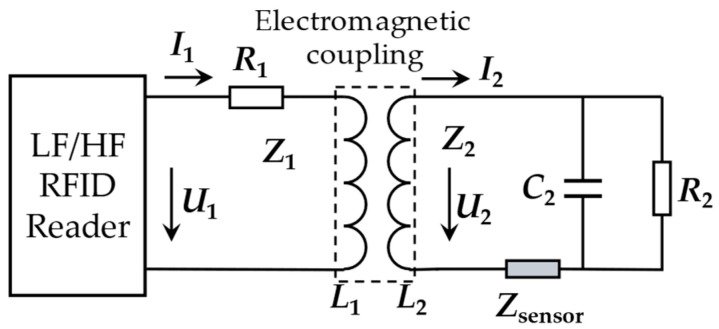
Equivalent circuit of LF/HF RFID tag.

The contactless electromagnetic transmission of time sequence, energy and data between the reader and tags is carried out through the antenna. The antenna is the bridge between the reader and tags, which is responsible for receiving and radiating RF signals. For LF and HF RFID sensing systems, the antenna is usually coils [31,32]. An equivalent circuit of the LF/HF RFID sensing system is pictured in Figure 3.

The system principle can be regarded as a transformer model, which is mainly composed of a series or parallel combination of inductors, capacitors, and resistors. The electromagnetic induction between the coils (*L*_1_, *L*_2_) realizes the transmission of energy and information. Typically, the tag coil acts as the sensing element. The equivalent resistance *R_L_* and inductance of the tag sensor are affected by the surrounding environment, which leads to the change of its characteristic impedance, and finally leads to the shift of the resonant frequency [31]:

(1) $$f=\frac{1}{2\pi \sqrt{C_{2}L_{2}}}.$$

**Figure 4:**
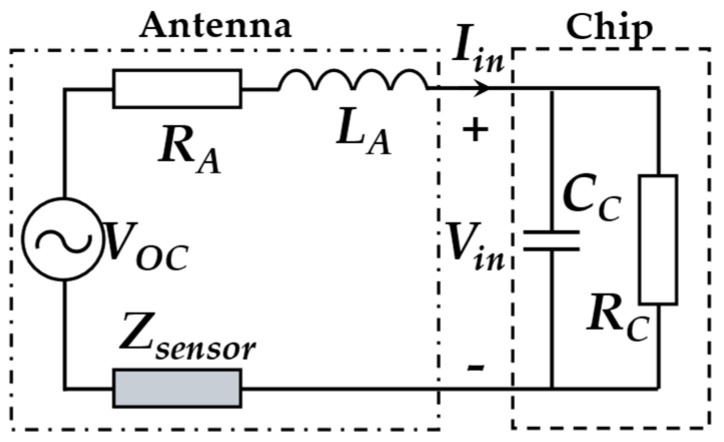
The equivalent circuit of UHF RFID tag.

For example, the electrical properties *C*_2_ and *L*_2_ are sensitive to temperature. The temperature content can be obtained with a solution model. Different from an HF/LF RFID sensing system, the energy and information exchanges between the reader and the UHF sensor tag belong to the backscatter coupling communication, which is based on the radar model. Figure 4 shows the equivalent circuit of the UHF RFID sensor tag. *V_OC_* represents the equivalent open circuit voltage of the antenna. *V_in_* is the input voltage obtained by the RFID chip. The impedance [33] of antenna and chip can be expressed as:

(2) $$Z_{A}\left(\varepsilon_{eff}\right)=R_{A}+jX_{A}\left(\varepsilon_{eff}\right)$$

(3) $$Z_{C}=R_{C}+jX_{C}$$

The transmission coefficient [12] between the chip and antenna can be calculated by:

(4) $$\tau \left(\varepsilon_{eff}\right)=\frac{4R_{A}\left(\varepsilon_{eff}\right)R_{C}}{|Z_{A}\left(\varepsilon_{eff}\right)+Z_{C}{|}^{2}}$$

where *ε_eff_* is the effective dielectric constant of the antenna substrate and the structures under monitoring. It can be noted from Equation (4) that *τ* reaches the maximum value when *Z_A_*(*ε_eff_*) and *Z_C_* are conjugate matched, i.e., *Z_A_*(*ε_eff_*) = Z_C_*. At this time, the corresponding frequency is the resonance frequency. The impedance of antenna is a function of *ε_eff_*. The environmental parameters change the electrical properties of the antenna, leading to its impedance variation and then resonance frequency shift.

Compared with WSN, the primary advantage of RFID is its unique identification ability. RFID eliminates wiring connections, which reduces installation time and maintenance costs. Furthermore, passive RFID is powered by the radio field generated by the reader without a battery. Low-maintenance or even maintenance-free RFID tags can even enable monitoring of structures throughout their life cycle. Hence these tags can be employed for large-scale SHM since they are wireless, low-cost, and battery-less. Various sensor tags are the core to obtaining structural health status. Detailed descriptions of different sensor tags will be presented in Section 4.

## 4. Designs and Applications of RFID Sensor Tags in SHM

The operating fundamentals of the RFID sensing system have been introduced in Section 3. According to these principles, different combination mechanisms of RFID and sensing technology result in various topologies of RFID sensor tags, i.e., commercial off-the-shelf (COTS) tag, antenna-based RFID tag, energy harvesting-based sensor tag and chipless RFID sensor tag. Each topology and its applications in SHM are discussed in more detail below.

### 4.1. COTS RFID Tag

Using the COTS RFID tag as a sensor tag is the most economical and accessible way, especially the COTS UHF RFID tags. These UHF RFID tags have the advantages of long read range, low cost per tag, high data transmission rates, and global standards. Commercial RFID systems can also be adopted directly in RFID-based SHM systems without redesigning components. Backscatter power, multipath effect and electromagnetic interference (EMI) shielding can be used as sensing mechanisms.

#### 4.1.1. Backscatter Power

**Figure 5:**
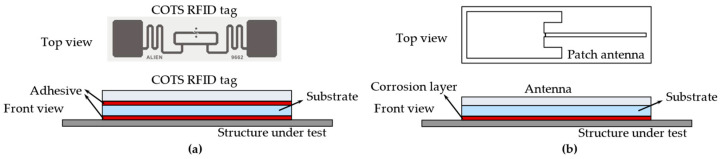
Diagram of RFID crack and corrosion sensor using backscattering: (**a**) COTS RFID tag as a crack sensor; (**b**) antenna-based RFID tag as a corrosion sensor.

The COTS UHF RFID tag exhibits different backscatter power signal capabilities when the environment near or within the tag is changed [34,35,36,37]. The Friis transmission equation gives the backscattered power of the tag [36]:

(5) $$P_{r}\left(\varepsilon_{eff}\right)=P_{t}{\left(\frac{G_{R}G_{T}\left(\varepsilon_{eff}\right)\lambda^{2}}{{\left(4\pi d\right)}^{2}}\right)}^{2}\cdot \tau \left(\varepsilon_{eff}\right)$$

where *P_r_*(*ε_eff_*) is the backscatter power, *P_t_* is the reader transmitted power, *G_R_* and *G_T_*(*ε_eff_*) are the gain of the reader antenna and tag antenna, *d* is the reading distance, *λ* is the wavelength of the carrier, *ε_eff_* is the effective dielectric constant of the tag substrate and the material of the structure under test, *τ*(*ε_eff_*) is the transmission coefficient calculated by Equation (4). In Equation (5), the backscatter power *P_r_*(*ε_eff_*) is a function of *G_T_*(*ε_eff_*) and *τ*(*ε_eff_*). Both *G_T_*(*ε_eff_*) and *τ*(*ε_eff_*) are sensitive to the environment (e.g., permittivity, permeability, conductivity, cracking, and corrosion in the structure under monitoring) near or within the tag since they are a function of *ε_eff_*. Usually, the backscatter power is reported in a logarithmic formation known as RSSI by the reader:

(6) $$\mathrm{RSSI}=10\log_{10}\left(P_{r}\left(\varepsilon_{eff}\right)\right)$$

where the unit of *P_r_*(*ε_eff_*) is milliwatt (mW), and the unit of RSSI is decibel relative to one milliwatt (dBm). This principle can be used for crack (or strain) detection when the tag is bonded (e.g., with epoxy or cyanoacrylate-based glue) on the structure, as shown in Figure 5a. The components of the crack sensor consist of a COTS RFID tag and substrate material, with each layer glued together. Since the percentage change in RSSI is relevant to the crack propagation [34], the percentage change in RSSI rather than the raw RSSI is used as a feature for cracks. The RSSI percentage change is calculated by:

(7) $$\Delta \mathrm{RSSI}=\left(\frac{{\mathrm{RSSI}}_{normal}-{\mathrm{RSSI}}_{crack}}{{\mathrm{RSSI}}_{normal}}\right)\times 100\%$$

where RSSI*_normal_* and RSSI*_crack_* are the RSSI of the uncracked and cracked states, respectively. The sensor in Figure 5a involves two aspects. One concerns the crack propagation from the structure through the substrate into the tag, causing the tag antenna to be severed. Another concerns the crack propagation causing strain in the substrate and tag antenna. This type of crack sensor has been tested, among others, for a single COTS tag and a 2D array [35,36] on metallic structures, which contributes to the development of RFID sensing systems for steel bridge health monitoring. In [38], COTS tags were used for passive material identification with different permittivity and crack detection in combination with multi-layer neural networks (MLNN).

The theory of backscatter power can also be used to detect cracks in non-metallic materials, such as concrete [22,39], and fiberglass combined material [40]. Using the backscatter power, the tag can detect propagation of ultra-high-performance concrete cracks [22,39]. With RFID tags integrated into an aeronautical fiberglass structure, deformation or breakage of the tag can be used to indicate its damage [40]. Some scholars explored COTS tags for conveyor belt crack monitoring [37,41,42,43] through RSSI measurements. They built a belt crack monitoring system with multiple antennas and developed a graphical user interface (GUI) for real time sensing display. The proposed system is capable of detecting cracks as narrow as 0.5 mm [37,42,43]. Machine learning (ML) algorithms (multilayer perceptron neural network and Naive Bayes classifier) were also adopted for crack detection and crack width prediction [41]. These measurement results show that the COTS tags have great potential for SHM.

Besides COTS tags, different antenna-based RFID sensor tags using the backscatter power principle have recently gained attention. Figure 5b illustrates a corrosion sensing model. The increase of the corrosion layer results in an increase in permittivity and permeability reduction, which leads to the transmission coefficient changing of the antenna. The miniaturized T-shape folded 3D antenna [11], dipole antenna [44] and a 3D antenna with an open-ended slot [12,45,46] were designed for corrosion detection. With the commercial RFID reader, these antennas successfully detect the various corrosion progressions.

#### 4.1.2. EMI Shielding Effect

EMI shielding is a technique used to protect equipment from environmental interference. EMI shielding is also used to prevent harmful electromagnetic waves being transmitted from the equipment to the environment. The EMI shielding and COTS RFID tags were exploited to form a simple corrosion monitoring system [47]. The communication between the reader and the tag is shielded by using metal film. Utilizing the metal film as a shielding layer hinders the communication between the reader and the tag. The metal film’s degree of corrosion will affect the EMI shielding effect. The power transmitted to activate a tag was minimal without EMI shielding. Once the metal films were attached to tags, none of the tags could be read. After the film is corroded, the shielding effect is reduced. The amount of power required to energize the tag was lower. Thus, a quantitative relationship was established between the tag backscatter signal strength and the corrosion rate. In addition, the tag response ratio, speed, and the minimum transmitted power to operate the tag could also be used as sensing variables [48,49]. In theory, cracks and corrosion of the shielding layer will weaken the shielding effect. However, RFID sensing systems cannot distinguish whether the shielding effect is weakened by cracks or corrosion. Further analysis is possible only in combination with the failure principle of the structure under test.

Different metal films, such as, aluminum [50], copper [50], and steel [47,48,49], were used as EMI shielding material. Compared with copper and aluminum, steel has the advantage of being closer to the material and corrosion rate of the structure under monitoring, which better reflects the actual corrosion status.

#### 4.1.3. Multi Path Effect

**Figure 6:**
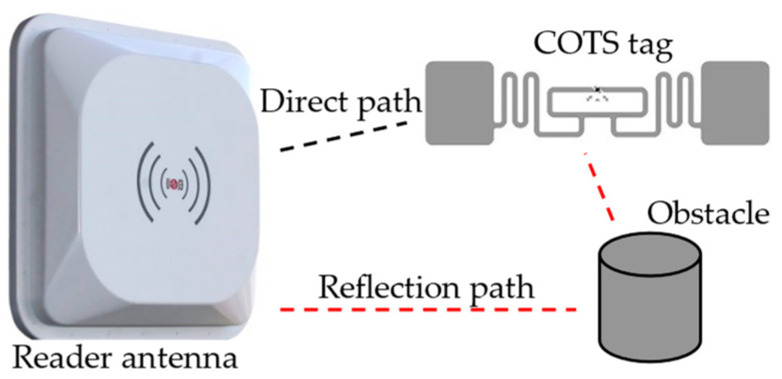
Multipath effect in RFID.

The RFID system’s multipath effect means multiple paths for the reader to read a tag. As shown in Figure 6, it can be divided into the direct path (reader antenna-tag-reader antenna) and reflected path (reader antenna-obstacle-tag-reader antenna). The time of arrival and phase of the received signal will differ for various signal paths. Furthermore, the object movement in the propagation path causes phase shifts and delays in arrival time.

**Figure 7:**
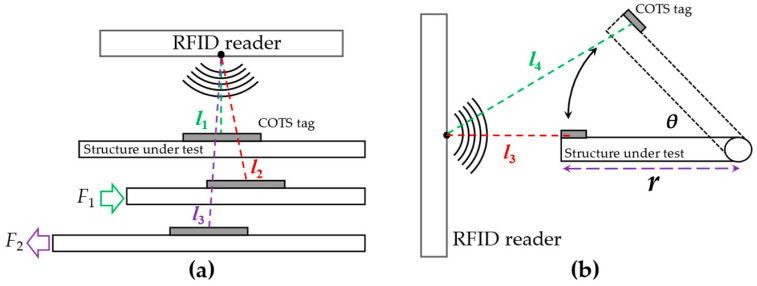
Schematic diagrams of RFID sensing system: (**a**) deformation model, (**b**) vibration model.

According to electromagnetic field theory, the relationship between the phase of the received signal and the reading distance can be expressed by:

(8) $$\varphi =-\frac{2\pi fd}{c}+\varphi_{A}$$

where *c* is the speed of light in vacuum, *f* is the frequency of reader carrier, *d* is the length of the signal path, and *φ_A_* is the phase shift generated by the reader circuit. It can be seen from Equation (8) that the difference in signal paths will change the phase of the received signal. Therefore, the movement of the tag (within a direct path) can be used to detect deformations and vibration (the tag is attached to the surface of the structure under test). The schematic diagrams of detection systems are displayed in Figure 7.

Suppose the reader antenna is an ideal point source. When a load strains the structure under test, the position of the COTS tag will change simultaneously (see Figure 7a). By comparing the phase shift of the tag, the deformation caused by force can be calculated [23]. In the vibration detection model, the COTS tag was attached to the position with the largest vibration amplitude of the structure under test (see Figure 7b). At this time, the phase shift caused by the vibration can be expressed as [51]:

(9) $$\left|\Delta \varphi \right|=\frac{2\pi f}{c}\cdot 2\left(l_{4}-l_{3}\right)=\frac{4\pi f}{c}\left(\sqrt{{\left(l_{3}+r\right)}^{2}+r^{2}-2r\left(l_{3}+r\right)\cos\theta }-l_{3}\right)$$

where *l*_3_ and *l*_4_ are the reading distance before and after vibration, *r* is the vibration radius, and *θ* is the angle of vibration. Notice the full signal path is twice the reading distance in backscatter communication.

For the vibration sensing system mentioned above, RFID tags are required to be attached to the structure under assessment. However, rotation and vibration can easily cause tags to be lost in long-term monitoring. Tags may also affect the operations of delicate devices, such as bearing damage and shaft misalignment. In addition to detecting vibrations using the direct path, a reflective path can also be designed for non-contact vibration detection with the same principle [52,53,54,55,56]. Although the multi-path principle can realize highly accurate and non-contact sensing with a COTS tag [56], achieving tiny amplitude vibration detection is challenging. Empirical researches show that reliable detection can only be achieved when the phase value shift is greater than 0.1 radian [57]. Otherwise, it will be submerged in thermal noise. Taking the vibration sensing at UHF band as an example: the wavelength is about 33 cm in 900 MHz. To achieve a phase change of 0.1 radian, the minimum vibration amplitude needs to reach 2.6 mm according to Equation (8). For the phase noise problem, RFID sensor tags that work within a higher frequency can be designed for a shorter wavelength. This solution can reduce the size of tag antenna, but higher frequency tags are not suitable for the protocol in the UHF RFID band.

#### 4.1.4. Discussion

Various COTS RFID sensor tags for SHM are listed and summarized in Table 3. The COTS RFID sensor tag has the advantages of simple structure and low cost. The sensing function can be realized without adding additional components based on the traditional RFID system. With adequately designs, sub-millimeter resolution can be reached [53]. However, this type of sensor tag also has certain limitations. For example, the critical principle of sensing with COTS tags is to detect the RSSI and phase information of backscatter signals, which is limited by the phase and power resolution of the reader. Secondly, the sampling rate (reading rate in RFID) of RFID readers is low. Assuming that the reader can read 100 tags per second, the sampling rate is 100 Hz. If we want to get the original measured signal without distortion, the frequency to be measured should be less than 50 Hz according to the sampling law. It will be lower if more than one tag exists. See Section 5 for a detailed description of these limitations.

### 4.2. Antenna-Based RFID Sensor Tag

The RFID tag comprises an antenna and an RFID chip (or integrated circuit, IC). Generally, the antenna is designed to conjugate with the IC at the resonance frequency. As mentioned in Section 4.1.1, any changes in the reactive or resistive part of the antenna will alter the loss of the matching network. Therefore, antennas sensitive to structural physical/mechanical parameters can be employed to design sensor tags. The following principles of antenna-based RFID sensor tags are introduced: switches, deformation, current path, and inter-antenna coupling.

#### 4.2.1. Switches

**Figure 8:**
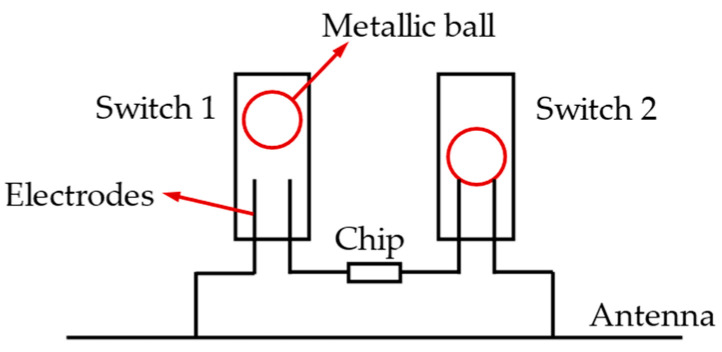
Schematic diagram of a vibration sensor tag with vibration-sensitive switches.

Sensing can be achieved by introducing switches sensitive to physical parameters between the RFID chip and the antenna. For example, RFID tags can measure vibration frequency by adding a switch. Figure 8 illustrates a vibration sensor tag with vibration-sensitive switches. The switch’s state (on or off) will determine the conduction between the antenna and the chip [62,63,64]. Therefore, the vibration frequency is related to the number of switching actions, which can be characterized by the reading times per second or the reading interval. Similarly, the maximum vibration frequency is limited by the reading rate of the reader.

#### 4.2.2. Deformations

The electrical length is closely related to the physical size of the antenna. According to the transmission line theory, the resonance frequency *f_R_* of a rectangular patch antenna can be calculated by [19,65]:

(10) $$f_{R}\left(0\right)=\frac{c}{2\left(L+2\Delta L\right)\sqrt{\varepsilon_{re}}}$$

where *c* is the speed of light in vacuum, *L* is the length of the rectangular patch, *ε_re_* is the effective dielectric constant, and Δ*L* is the compensation length. It can be seen from Equation (10) that the resonance frequency is correlated with *L*. If the antenna is attached to the surface of the structure, its resonant frequency will change with the deformation of the structure. This mechanism can be developed to detect structural deformation or strain, that is:

(11) $$f_{R}\left(\varepsilon \right)=\frac{c}{2\left(1+\varepsilon \right)\left(L+2\Delta L\right)\sqrt{\varepsilon_{re}}}=\frac{f_{R}\left(0\right)}{1+\varepsilon }\approx f_{R}\left(0\right)\cdot \left(1-\varepsilon \right)$$

where *ε* is the strain in the length direction of the antenna. Hence, the resonance frequency shift (RFS) caused by strain is calculated as:

(12) $$\Delta f=f_{R}\left(\varepsilon \right)-f_{R}\left(0\right)\approx -f_{R}\left(0\right)\cdot \varepsilon$$

Equation (12) can be regarded as the strain sensing mechanism of antenna-based RFID sensor tags. The RFS has an approximately linear relationship with strain. The existence of stress and stress concentration will severely impact the mechanical properties, corrosion resistance and fatigue resistance of structures or components. In order to ensure the safety of major mechanical equipment and key components, it is promising to obtain the stress-strain distribution of the mechanical structure through this method. The rectangular patch antenna is the most common way to design a strain sensor. Folded patch antenna [19], slotted patch antenna [66], inverted-F antenna [16], and meandered half-wave dipole antenna [24] were explored accordingly. However, these sensor tags could detect strain with its direction parallel to the rectangular patch. There will be a fatal error when an angle exists between the length direction of the rectangular patch and the strain direction. This is because the width of the rectangular patch is also deformed according to Poisson’s ratio, which will also affect the resonance frequency. Some scholars studied the relationship between the resonance frequency and horizontal/vertical strain [14,67] and the influence of transverse strain on the resonance frequency of the patch antenna. A novel design was reported in [18] to realize omnidirectional strain detection. Short stub feed patch antennas with different sensitivities in the length and width directions were combined to form a sensor tag array for strain magnitude and direction detection. The feasibility of this method has been confirmed through experiments and numerical simulations [18]. However, further studies are needed for the wireless performance of the array. These studies lay the foundation for a more comprehensive assessment of structural stress states.

#### 4.2.3. Current Path

**Figure 9:**
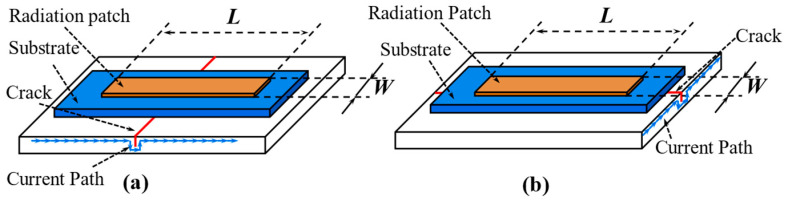
Effects of cracks on current paths. (**a**) a crack parallel to the width of the rectangular patch leads to an increase in current path along the length of the rectangular path. (**b**) a crack parallel to the length of the rectangular patch leads to an increase in current path along the width of the rectangular path.

The current path principle is usually employed to crack detection with the antenna-based RFID sensor tag. Taking the rectangular microstrip patch antenna as an example, the working principle of the RFID crack sensor is introduced. As shown in Figure 9, the surface of the structure was seen as the ground plane of the patch antenna. A specific frequency electromagnetic resonant cavity is formed between the radiation patch and the metal ground plate. The rectangular patch antenna includes two basic radiation patterns (TM_10_ and TM_01_), and the current directions are different under different radiation patterns. The generation of cracks will increase the path of the current distribution, increasing the antenna sensor’s electrical length in this radiation pattern. For example, a crack parallel to the width of the rectangular patch results in increased current paths along the length of the rectangular path. The resonance frequency *f*_10_ corresponding to the TM_10_ radiation mode is affected, while *f*_01_ corresponding to the TM_01_ is not affected.

Quantitative research on cracks with different antennas using this principle is a hot spot, such as crack length, width, depth, and direction. With a circular patch antenna as a crack sensor, a linear relationship between the resonance frequency and crack parameters, i.e., depth, length, width, and cross-sectional area, has been obtained [8]. A rectangular patch antenna was developed for crack depth and length detection with mm resolution [9,68,69,70,71]. Crack localization [69] and multi-tag coupling [9,70] were further investigated. A 3D antenna was designed in [45] for crack depth evaluation. A mm resolution can be achieved by extracting the phase and amplitude from backscatter signals and combining them with the kernel principal component analysis (PCA) method. Although use of antenna-based sensor tags shows exciting possibilities in crack monitoring with the current path principle, it still faces many challenges. For example, the position of the surface crack will influence the consistency of the tag’s response. Besides, the crack detection results can be affected by the simultaneous presence of multiple cracks in the area covered by the sensor tag. Moreover, the response of the tag should be studied when multiple parameters of the crack are changed, e.g., simultaneously changing the depth and width of cracks. In other words, multi-crack monitoring and multi-parameter decoupling methods should be further investigated.

#### 4.2.4. Inter-Antenna Coupling (Coupled Tags)

**Figure 10:**
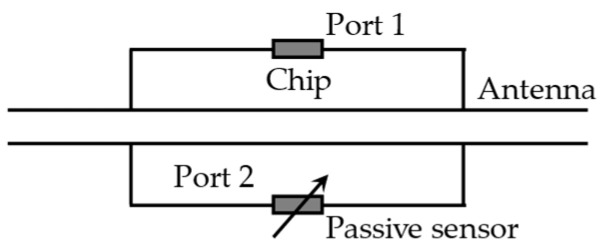
Two-port sensor tag.

Caizzone et al. [72] proposed a scatterer with two ports. One port was connected to the UHF RFID chip, while another port was connected to the variable load made by the transmission line, as shown in Figure 10. The change in transmission line impedance will affect the solid electromagnetic coupling between the two ports, reflected in the amplitude and phase of the backscatter signal. A crack mouth opening displacement gauge was made based on the dual-ports scatterer [10]. Experimental results show that the gauge has a minimum sensitivity of 16 °/mm (phase shift). Another inter-antenna coupling-based crack sensor was reported in [73,74]. One tag was placed on each side of the crack. The phase and amplitude of the backscatter signal will be dependent on the mutual position between tags, which is affected by the evolution of the crack. However, the sensing technique using phase information usually needs a fixed reading distance.

#### 4.2.5. Discussion

Various antenna-based RFID sensor tags for SHM are listed and summarized in Table 4. The difference between antenna-based RFID sensor tags and COTS tags lies in the antenna design. As a sensing element, the antenna is the core of designing tags, such as the anti-metal characteristics, miniaturization, sensitivity, and robustness. Tags in this topology can still use commercial RFID chips. Hence, commercial RFID readers are also suitable for this situation. However, most tags using the principles of deformation, current path, and backscattering are measured and converted into impedance parameters, which cannot be directly measured by the reader. It needs to perform frequency and power sweep in its working frequency band, which requires higher requirements for the reader and is time-consuming. Furthermore, the reduction in sensing (or reading) distance reduces the coverage of an RFID sensing system compared to COTS RFID sensor tags.

### 4.3. Digitally Integrated RFID Sensor Tag

#### 4.3.1. Architecture of Digitally Integrated RFID Sensor Tag

**Figure 11:**
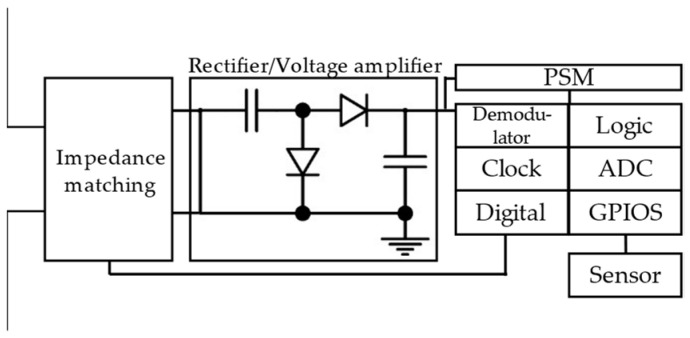
Schematic of digital integrated RFID sensor tag.

Compared with COTS RFID tags and antenna-based tags, digitally integrated RFID sensor tags have digital circuits. The tag is powered by the radio field generated by the reader. Advanced power supply management (PSM) strategy is integrated into the tag. The sensor and microcontroller are activated after enough energy has been harvested to support their work. The schematic of the digitally integrated RFID sensor tag is given in Figure 11. The RF energy from the RFID reader will be captured and converted to AC power through the antenna. The impedance matching circuit ensures a highly efficient AC flow. Then the AC power is converted to DC power by the rectifier/voltage amplifier. PSM is responsible for storing DC energy and converting it into a stable power supply for logic circuits and sensors.

There are two design methods for digitally integrated RFID sensor tags. One is to adopt discrete electronic components, such as wireless identification and sensing platform (WISP) [75,76] and battery-less wireless sensor tag (BLWST) [77]. WISP is an RFID platform for identification and sensing, which is compatible with the ISO-180006C standard. The second is to use RFID sensor chips, e.g., Rocky100 [58] and SL900A [78]. A bending strain sensor based on Rocky100 [58] and an absolute force sensor based on SL900A [78] were explored, and their reliability was verified.

#### 4.3.2. Discussion

Different digitally integrated RFID sensor tags are listed and summarized in Table 5. The tag transmission signals are digital, and their accuracy and anti-interference ability are more significant than antenna-based sensor tags. However, digitally integrated RFID sensor tags work discontinuously with limited energy, and their communication distance will be influenced too. The sensor activity time and reading distance are limited by the energy acquisition method, energy harvesting efficiency, and the power consumption of the sensor. The main challenge of digital integrated RFID sensor tags is to ensure the correct operation of digital circuits under energy-limited conditions with suitable accuracy. Hence, proper energy storage and utilization methods are essential. Effective solutions also include improving the antenna gain and radiated power (maximum 36 dBm). Multiple power sources have been investigated to improve the read range and sensing performance, such as assistant battery [15,79,80,81], solar power [80,81], RF signal from TV stations [82] and vibration energy harvester [83,84].

### 4.4. Chipless RFID Sensor Tag

#### 4.4.1. Architecture of Chipless RFID Sensor Tag

**Figure 12:**
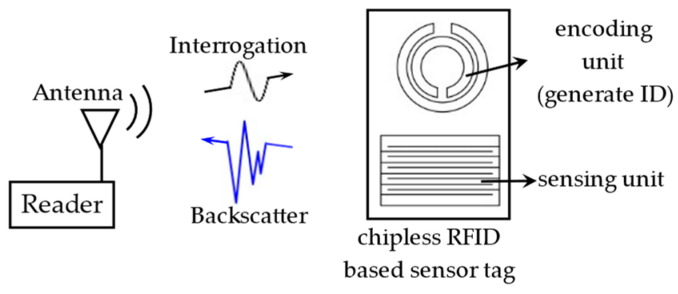
Block diagram of chipless RFID based sensor system.

The block diagram of the chipless RFID-based sensor system is shown in Figure 12. Chip-less RFID does not require integrated chips or digital circuits, making the RFID system simpler and more convenient.

Chipless RFID sensor tags are divided into two parts: an encoding unit and a sensing unit. There are various coding methods: shape-based [88], time domain, frequency domain, amplitude/phase domain [89], and hybrid [90]. The principle and comparison of different coding methods are shown in Table 6. It can be seen that capacity and density are the main problems faced in designing coding units.

**Figure 13:**
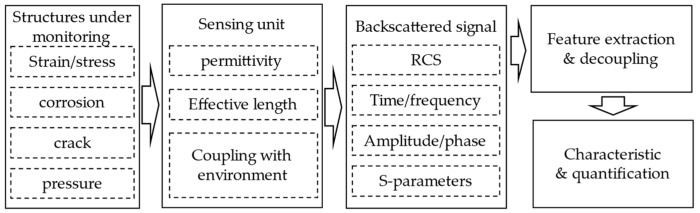
Sensing procedures of chipless RFID sensor tags.

The operating principle of the sensing unit is similar to the antenna-based RFID sensor tag, such as the electrical length change caused by deformation [26], current path [91], and impedance coupling [92]. These will result in a change in the characteristics of the backscatter signal, such as RCS [20,21,93], S-parameters [94], time or frequency domain [95,96], and amplitude or phase domain [29,97]. The sensing procedures of chipless RFID sensor tags are illustrated in Figure 13.

#### 4.4.2. Discussion

Various chipless RFID tags have been developed for sensing, for example, a circular microstrip patch antenna (CMPA) for damage and strain sensing. Typical chipless tags for SHM are listed in Table 7.

Chipless RFID sensor tags are inexpensive and easily printed on various substrates given the absence of chips and circuits. The tags can be simple, printable, and durable in harsh environments. Despite the simple structure of chipless RFID sensors, there are still many problems to be solved before large-scale practical SHM application:

(1) Coding capacity: there is no chip inside the tags. The coding methods and capacity are the primary problem of RFID technology;

(2) Interrogation distance: the interrogation distance of chipless tags (Table 7) is shorter than chip-based RFID sensor tags, which remains a challenge for the following aspects: The first one is the low radar cross section reflects from tags. The second is that environmental clutter reflections are stronger than the tag response.

(3) Robustness: chipless RFID sensors communicate with analogue signals, and sensing signal reading is susceptible to interference due to multi-path and environmental effects. In addition, a specific reading direction is required (since these tags do not have polarization independence, the angle of the incident wave of the interrogation signal must be kept constant). In other words, the placement angle of the sensor tag is fixed, and any angle shift will affect the detection and recognition of the tag).

(4) Reader: there is no standard communication protocol. Most applications use a vector network analyzer (VNA) as a reader, which has high cost and low flexibility.

## 5. Technical Challenges and Solutions

Compared with the limitations of distributed sensors requiring wiring and battery power, the RFID sensing system provides a cost-efficient, battery-less, and wireless solution for SHM. The RFID sensing system has made significant progress in SHM, but some challenges hinder its further applications in large-scale real-world structures. It is expected to form a smart skin to achieve high-granularity monitoring for large-scale structures, especially for metal structures with complex profiles. However, the rigid dielectric substrate, bulk, and performance of RFID sensors on the metal structure surface limit their industrial applications. In addition, for the sensing system, there are still problems such as real-time performance, the balance of communication and sensing, and system construction. Therefore, this section will discuss these technical challenges and solutions in the context of industrial applications. These technical challenges and solutions may be summarized as follows:

### 5.1. Antenna Design

Antennas are essential components in the RFID sensing system, which has the role of communication and sensing. However, antennas are subjected to interference when mounted on metallic surfaces. Moreover, the spatial sensing resolution depends on the antenna’s dimensions. Hence, improving anti-metal performance and antenna miniaturization are critical issues in RFID sensing systems.

#### 5.1.1. Anti-Metal Performance

**Figure 14:**
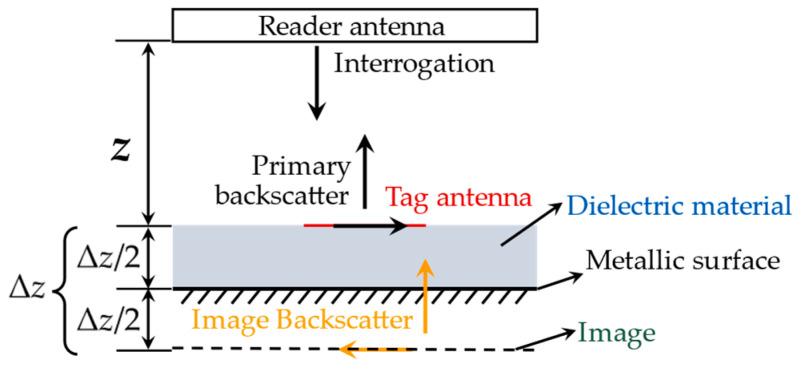
Effect of metallic surface on RFID tag antenna (image theory).

SHM is mainly applied in metal structures, such as oil derricks, pipelines, and aircraft. Affected by metal boundary conditions, metal surfaces will reduce the gain and destroy the antenna’s impedance matching and radiation pattern [28]. In particular, COTS RFID tags are designed for communication and identification. If the COTS RFID tags were used for metal surface sensing, the reading distance would be sharply reduced [44]. The performance of RFID antennas on metallic surfaces can be explained by image theory (see Figure 14). The metallic surface can be viewed as a ground plane when the tag antenna is close. The backscatter power *P_r_*(Δ*z*) [34] received by the reader antenna can be described by:

(13) $$P_{r}\left(\Delta z\right)=\exp\left(-j\beta_{air}z\right)\left(I_{tag}+I_{image}\exp\left(-j\beta_{material}\Delta z\right)\right)$$

where *β_air_* and *β_material_* are the phase constant of air and substrate, respectively; *I_tag_* and *I_image_* are the primary and image backscatter of the tag antenna, which has a relationship of *I_tag_* = −*I_image_*. Δ*z* is the effective distance between the tag antenna and its image, and *z* is the read distance. When the tag is placed directly on the metallic structure surface, e.g., Δ*z* = 0, the *P_r_*(Δ*z*) will be zero, according to Equation (13). Hence, the tag cannot be interrogated by a reader. The amplitude of backscatter power reaches a maximum when the reflected wave of the image has a 180° phase shift. Namely, Δ*z* is half of a wavelength. Accordingly, an RFID tag should have an effective quarter wavelength distance from the metallic surface. Based on the above analysis, the easiest way to ensure anti-metal performance is to adjust the distance between the tag and the metal surface. Polystyrene foam was used as a substrate material in [34,35,36] to maximize the COTS tag’s backscatter power. Inevitably, the overall height of the tag is increased, which is inconvenient for sensing purposes.

Recent studies have shown that adopting a microstrip patch antenna is the most popular approach to improving anti-metal performance. A patch antenna comprises a layer of metallic patch, substrate layer, and metallic ground plane [103]. The surface of the metallic structure under test can be regarded as the specific ground plane of the antenna and thus can be effectively used for a metal environment. For example, rectangular [23,68,69,104,105,106] and circular patch [13,25,107,108] antennas with different structures are being developed for strain [23,25,104,105,107], crack [68,69,106,108,109], and corrosion [13] sensing of metal structures. In addition, developing an absorbing material [110] and utilizing specific electromagnetic band gap structures [111] are effective methods to solve the anti-metal problem.

#### 5.1.2. Miniaturization

In practical application, the miniaturization of antennas enables a denser distribution of sensor tags within the same monitoring area [70]. Meanwhile, the reduction of antenna dimensions could increase the current in a specific area and thus improve the crack and corrosion sensitivity and resolution [11,45]. Several miniaturization methods can be found in recent investigations, such as short circuit vias [18], folding [11], slotting [23] and substrate with high dielectric constant [112]. From Equation (10) it can be concluded that the length of antenna and the effective dielectric constant has a contrary relationship within a frequency point. In other words, the antenna size is reduced at the same frequency due to the high dielectric constant substrate used. Slotting and folding can increase current path on the surface of antenna, thereby reducing the size of antenna. A miniaturization patch antenna using meandering and folding methods was developed in [11] for corrosion monitoring, and a slotted patch antenna was proposed in [66] for stress concentration detection. Besides, the quarter wavelength patch antenna with shorting vias [18] and the planar inverted-F antenna (PIFA) [16] seem to be interesting ways of achieving antenna miniaturization.

Ensuring the communication and sensing capability of the antenna while improving anti-metal performance and reducing its size deserves special attention, because these operations usually bring high loss and low gain.

### 5.2. Multi-Parameters Detection

Recent RFID sensing systems for SHM have demonstrated their feasibility for obtaining data on the structural health status, such as strain, crack, corrosion, and vibration, as discussed in Section 4. The responses of RFID under single variables such as strain magnitude, crack length, width, and corrosion time were studied. Unfortunately, a single parameter cannot reflect complete information about the structural health status. For example, the crack length, width, depth, location and orientation are all important parameters to evaluate the health state of structures in crack sensing applications. Alternatively, strain direction and magnitude are both important parameters for evaluating the strain states affecting the strain sensing system. In addition, sensor tags are affected by environmental factors such as temperature. Then, additional parameters may be required for temperature compensation. In conclusion, multi-parameter detection systems are urgently needed to provide a comprehensive, robust assessment of structures.

A multi-parameters system can obtain multiple sensing parameters with a single sensor or integrate different sensors to obtain each parameter separately. A crack and temperature sensor tag was developed in [92], providing stable precision in harsh climates. In [113], a dual-band circular microstrip antenna was explored for strain omnidirectional detection, which achieved strain amplitude and direction detection within a single antenna sensor compared with the antenna array in [18]. T. Wang [114] proposed a dual-tag sensor to eliminate the common mode interference through differential backscattering signals [114]. Multi-sensory systems were also investigated in [76] for temperature and acceleration measurement. The acquisition of multi-sensing parameters avoids the uncertainty and contingency of single-parameter sensing, which is more comprehensive and robust.

It is worth noting that the multi-parameters sensing system places higher demands on the design of RFID antenna tags and low-power technology while improving performance. In addition, the decoupling between multiple parameters and effective feature extraction methods also needs attention.

### 5.3. Low Sampling Rate of RFID Sensing System

SHM is a real-time and long-term system that continuously monitors the status of structures. However, the RFID sensor tag has a low sensing stream, which is restricted by the inherent properties of the sensing system, specifically in the following aspects:

(1) The performance of RFID reader. For COTS and antenna-based RFID sensor tags, the sensing variables are usually the number of reading times, reading interval and the phase of the received signal. In this case, the reader obtains one sampling datum after each reading. Limited by the reading rate and multi-tags inventory performance of RFID readers, there is an upper limit on the sensing sampling rate. According to the Nyquist sampling law [57], a signal must be sampled at more than twice the highest frequency component of the signal. For example, the maximum read rate of Impinj fixed readers is 1100 per second. The sampling rate is 1100 Hz. There are *N* tags in the monitoring area. If we want to get the original measured signal without distortion, the frequency to be measured should be less than 550/*N* Hz.

(2) Sensing strategy. One sensing datum can be obtained only by multi-readings when the resonance frequency is taken as a feature. According to the impedance matching principle, the frequency point corresponding to the lowest turn-on power or the frequency point corresponding to the highest RSSI is the resonant frequency. The turn-on power or RSSI of each frequency point is obtained through frequency and power scanning in the frequency band. In this case, the low sampling rate of the sensing system is due to the measurement strategy of obtaining a single data point through multiple readings. For example, a pseudocode of the reader used to read the crack sensor tag is shown in Table 8. A set of queries [59] is required for one resonance frequency point, which leads to the low sampling rate of RFID sensing data.

(3) Limited energy. For digitally integrated RFID sensor tags powered by the radio field generated by the reader, the tag should be worked with limited energy. In order to avoid start-up failure, tags should be powered after enough energy has been harvested to support its initialization [77].

Many scholars have made efforts to address these issues in different novel ways. For example, compressive reading was proposed to address the sample rating problem [51]. However, this method is only suitable for periodic signals. By extracting the sensing information from the physical layer rather than the application layer of RFID, the low sampling rate of the RFID sensing system can be relieved [57,61]. Li Ping et al. explored the harmonic backscattering-based vibration sensing system called *Tagsound* to solve the lower reading rate [57,61]. *Tagsound* used high-order harmonics generated by the nonlinearity of the rectifier in the UHF RFID tag. Taking advantage of the harmonics, the upper bound of perceptible frequency is significantly increased. Harmonic sensing can increase the sampling rate of RFID sensor systems. However, additional harmonic sniffer readers are required compared with a traditional RFID system.

### 5.4. Communication and Sensing

For antenna-based RFID sensor tags and chipless RFID sensor tags, a key challenge for an antenna is the balance between communication and sensing [27,28]. Gain and sensitivity are primary considerations to characterize the communication ability and sensing ability of the antenna, respectively. From a communication standpoint, the antenna should have a low Q-factor to ensure high gain and bandwidth [115], but this will reduce the sensitivity [68]. From the sensing perspective, the antenna should have a high Q-factor to obtain ideal sensitivity [116], but the gain of the antenna is decreased due to extraordinary losses [117]. Consequently, enhancing robustness while maintaining antenna gain with considerable sensitivity is challenging.

Separating the sensing and communication units of the antenna sensor is an effective solution to balance their performance. An electromagnetic-induced transparency-inspired antenna was designed to address this issue [6,7]. A low Q-factor split-ring resonator render is responsible for communication, and two back-sited U-shaped strip resonators with high Q-factor are designed for sensing. The antenna sensor achieves high-sensitivity crack characterization at a reading distance of 1 m [6]. A high Q-factor circular three-arm element was designed for high sensitivity corrosion detection (a sensitivity of 13 MHz at the standard UHF band). A parasitic element was added to the antenna to improve the gain of the antenna, which enabled the extended reading distance (up to 2 m) while maintaining the sensor sensitivity.

### 5.5. Chipless RFID Sensing System

A VNA usually acts as the reader in the chipless RFID sensing system. As a general-purpose measurement platform, VNA has high costs and low programmability and is not suitable for real-time sensing in industrial applications [105]. A strain detection system based on universal software radio peripheral (USRP) was introduced in [105,118,119]. The software radio development platform has better flexibility, compatibility, and openness than VNA, which provides a way to widen application scope and reduce costs. Based on this platform, advanced machine learning algorithms can be integrated to realize real-time and intelligent strain detection.

Reading range is a further practical issue. The backscatter signal is weak, and its interrogation distance (see Table 7) is shorter than that of chip-based RFID sensor tags. The current short distance may not be suitable for large-scale applications in SHM. Increasing the gain and directivity of the reader antenna can improve the reading distance but increase the volume of the reader [100,120]. The cross-polarization reading and depolarizing sensor can reduce inevitable environmental reflections [95]. This may be a possible way to improve the reading distance, but more experimental verification is needed.

### 5.6. Printable and Flexible Sensor Tags

The aforementioned damage sensors (crack and corrosion) have achieved impressive performance, but their substrate materials are mostly rigid (e.g., FR-4). Many metal structures have non-planar surfaces, such as pipes and aircraft skins, which pose new requirements for the design of RFID damage sensors.

In recent years, with the development of printed electronic technology, it has been widely used in the field of flexible sensing. Using this technology, tag circuits can be fabricated on various flexible substrates (such as paper-based, cloth-based, high polymer and other flexible materials). This approach may enable design of RFID sensor tags with excellent performance. Nappi et al. [121,122,123,124,125] proposed inkjet printable space filling curves (SFC). Combining SFC (e.g., Gosper SFC [121]) with RFID technology can enable detection of small defects (as tiny as 0.6 mm) within a large surface. Multiple SFC cells were arranged to form the space filling surface. Space filled surfaces can be applied to metal (implanted metal prostheses crack [122,125]) and non-metal objects (3D-printed ABS pipeline [123,124]). In addition, various other novel flexible sensor tags have been developed, such as SRR resonators on polyimide films [126] and 3D printable Ninjaflex flexible substrates [127].

### 5.7. Structural Health Prognostics

SHM based on RFID can obtain the real-time operating status of devices under test passively and wirelessly. Combining condition monitoring data with advanced algorithms [128,129] to accurately estimate the structural health state and remaining useful life is a development of SHM, which can be described as structural health prognostics (SHP).

In [87], a deep learning approach was introduced to monitor the wind turbine planetary gearbox condition. Deep learning algorithms such as chaotic quantum particle swarm optimization, deep belief network, and the least-squares support vector machine are used for fault classification and prediction. The proposed approach shows great potential for wind turbine health prognostics.

## 6. Conclusions

SHM is necessary to ensure the reliable operation of large structures such as oil derricks, railway, pipelines, wind turbines and transformers. Sensing systems based on RFID show great potential in SHM due to the advantage of long reading range, and being battery-free and wireless. This work presents the progress of the RFID sensing system for SHM applications. RFID sensor tags are classified based on their working mechanisms, i.e., COTS, antenna-based, digitally integrated, and chipless RFID sensor tags. COTS tags and commercial RFID readers can be directly used for SHM without adding additional components. However, COTS tags are designed for non-metal object identification; the metallic boundary condition will influence their radiation pattern. The progressiveness of antenna-based RFID sensor tags is reflected in their compatibility with conventional RFID readers. Researchers can flexibly design the antenna’s sensitivity, volume, and anti-metal performance according to the characteristics of the SHM. Nevertheless, the sampling rate is low due to the sensing strategy. Digitally integrated sensor tags have a low power consumption sensor, which has stronger robustness and anti-interference, but the reading distance is reduced in fully passive mode. Chipless RFID sensor tags are inexpensive and easily printed on various substrates without chips and circuits. Conversely, the complexity of RFID readers is increased due to missing the chip and circuits. The reading range and robustness of chipless sensor tags in real-worlds application are still ongoing research areas.

A bibliographic survey was carried out to explore the main applications relevant to SHM. Based on this, techniques, challenges and solutions were presented, including antenna design, multi-parameter detection, communication and sensing, and the sampling rate of the RFID sensing system. Advances in flexible materials and printable technologies have paved the way for low-cost passive large-scale structural health monitoring. With the fusion of fault diagnosis, classification and prediction algorithms, the technical scope of RFID sensor tags extends to structural health prognostics. Thus, there are many possibilities for further improvement in the design and implementation of RFID sensor tags.

## Figures and Tables

**Table 1:** Detailed information of retrieval and selection.

Database	Web of Science
Topic for retrieval	(radio frequency identification *or* RFID) *and* (sensing *or* sensor) *and* (structural health monitoring *or* SHM)
Publication years	2017–2022
Document types	exclude patents
Categories for refinement	strain or stress, crack or corrosion, and vibration in SHM

**Table 2:** Types of RFID operating in different frequency ranges [30].

Frequency Band	LF (30~300 kHz)	HF (3~30 MHz)	UHF (300~3000 MHz)	
Primary frequency	125~134 kHz	13.56 MHz	433 MHz; 860~960 MHz; 2.45 GHz	
Power source	passive (RF energy)	passive (RF energy)	passive (RF energy)	semi-passive/battery
Read Range	shorter than 10 cm	shorter than 30 cm	shorter than 25 m	longer than 30 m
Applications	animal tracking, access control; car key-fob; application with high-density liquids and metals	identification (ID) cards; near-field communication (NFC) application; library books	supply chain tracking; manufacturing; pharmaceuticals; electronic tolling; inventory tracking; race timing; asset tracking	vehicle tracking; auto manufacturing, mining; construction, asset tracking
Pros	high performance near water and metal; global standards	larger memory options, global standards; NFC global protocols	long read range; low cost per tag; wide variety of tag sizes and shapes; global standards; high data transmission rates	very long read range; lower infrastructure cost (vs. passive RFID), large memory capacity; high data transmission rates
Cons	low data rate; short read range; limited quantity of memory	low data rate; short read range	high equipment costs; moderate memory capacity; high interference from metal and liquids	shipping restrictions (due to batteries); complex software may be required; high interference from metal and liquids; few global standards

**Table 3:** COTS RFID sensor tags for SHM.

Tag	Sensing Parameters	Sensing Variables	Sensitivity	Application Notes	Ref.
IMPINJ H47 tags	bending stress assessment	active power	n/a	bending stress assessment; low sampling rate: measuring active power in full frequency (902–928 MHz)	[58]
Alien Technology ALN-9662 short Inlay tag	steel structures crack	RSSI	n/a	reading distance: up to 15 ft; 2D tag array assessment; multiple tag coupling effect needs to further studied	[34,35,36]
various COTS tags	slot cracks of carbon steel and stainless steel	power/phase, machine learning	accuracy of crack feature extraction: 84.4% in width and 78.7% in depth.	reading distance: 1.8 m; RFID defect sensing integrated with machine learning techniques; future efforts required for quick response	[38]
commercial tag (n/a)	crack depth	RSSI	n/a	reading distance: 35–40 cm; maximum crack depth estimation error of 0.1 mm (stainless steel sample); less accurate for ferromagnetic material	[59]
Texas instrument HF tag (RF-HDT-DVBB-N2-TAG)	corrosion time	transient response	n/a	early-stage corrosion monitoring; the number of steel samples is relatively small	[60]
ALN-9654 from Alien technologies with a Higgs-3 chip	corrosion	RSSI	0.4 dB/mm	reading distance: 15 cm; ability to follow the loss of thickness of metals; only suitable for indoor application	[44]
commercial RFID tags	corrosion	RSSI/threshold transmitted power	n/a	reading distance: 0.6 m; simple and cost-effective wireless corrosion monitoring sensor; reference tag required	[47]
AZ9662 H3 commercial RFID tags	composite structures internal damage detection	n/a	n/a	simple damage detection method; basic research state	[40]
Alien technology ALN-9662 short Inlay tag	concrete cracks	RSSI	n/a	reading distance: 88.9 cm; artificial neural network-based cracks monitoring; Low-cost tags	[22,39]
Alien tags	vibration	backscatter power	vibration period relative error of 0.03% and mean accuracy of 0.36 ms	reading distance: 1.5 m; compression reading algorithm; limited by the minimum reading rate; limited by multi-tags performance of reader	[51]
commercial tags	vibration	phase	frequency up to 400 Hz with a mean error of 0.2%	reading distance: 1.5 m (default); contactless vibration sensing multiple devices monitoring capability with a single tag; larger error for nonmetallic materials	[52,54,55]
COTS RFID tags (ImpinJ and Alien)	vibration	base band signal	mean error: 0.37 Hz (<100 Hz); mean error: 4.2 Hz (~2500 Hz)	reading distance: 0.4 m; detection capability of high-frequency and tiny mechanical vibrations; not limited by the reading rate; environmental interference	[57,61]
COTS tags	vibration	phase	vibration amplitude: 0.5 mm	reading range: 1.5 m; contactless vibration sensing; sub-millimeter vibration accuracy; limited by multi-target sensing (coupling effect)	[53]

**Table 4:** Antenna-based RFID sensor tags for SHM.

Tag Design	Sensing Parameters	Sensing Variables	Sensitivity	Application Notes	Ref.
the microstrip antenna and FR4 substrate	strain of metal structures	phase	mean phased difference about 12°/2 cm	good anti-metal performance; commercial reader; low sensitivity (phase shift); only suitable for horizontal shape changes	[23]
planar inverted-F antenna and CTC13001 chip on N9220 substrate	strain of aluminum sheet	reading range	550 Hz/με	reading range: >1.7 m; miniaturized antenna; measuring uniaxial strain; low sensitivity	[16]
meandered dipole antenna and Higgs-4 chip on RO4350 substrate	strain measurement on metals	RSSI	n/a	reading range: 0.16 m; reusable: the sensor does not deform during specimen loading; only suitable for uniaxial strain	[17]
short stub feed patch antenna on FR4 substrate	strain of metal structures	s-parameters	horizontal strain: −873.91 Hz/με; vertical strain: 57.28 Hz/με	strain magnitude and direction detection; RFID sensor tags array; wire interrogation; wireless performance needs to be verified	[18]
folded-patch antenna and SL3S1013 chip on RT/duroid 6202 substrate	strain and crack sensing	interrogation power	−599 Hz/με	interrogation distance: 0.9144 m in fully passive mode; better thermal stability; multi-reading required for a single strain data	[15]
3D-antenna	crack depth and corrosion progression	backscatter power/phase	n/a	reading distance: 1 m; feature extraction and selection through PCA; high antenna profile (16 mm)	[12,45,46]
electromagnetically induced transparency inspired antenna	slot crack depth and width	turn on power	crack depth: 2.73 MHz/mm^2^; crack width: 2.75 MHz/mm^2^	reading distance: 1 m. performance balance between sensing and communication; the relative position of the crack may influence the sensitivity	[6,7]
rectangular patch antenna on FR4 substrate	crack depth and length	read threshold transmitted power	n/a	sensing the change of surface crack with mm-resolution; reducing the disturbance of crack location with miniaturized sensor tag; multi-crack detection problem and dual-tag coupling effect need to further studied	[9,68,69,70,71]
T-shape folded antenna and Monza-4 chip	corrosion exposed time	threshold power	45 kHz/μm	reading distance: 0.66 m; folded antenna can reduce the dimension of the antenna but decrease the sensitivity and peak gain	[11]
two-port UHF RFID tag	crack opening displacement	phase	Phase shift: 16 °/mm. Maximum measurement range: 5 mm	reading distance: longer than 1.5 m; high resolution and sensitivity	[10]
antenna, tilt/vibration sensors, and RFID chip	vibration	read interval or read rate	n/a	reading distance: 0.5–1 m; simple structure; limited by the reading rate; suitable for vibration with low frequency and large amplitude	[62,63,64]

**Table 5:** Digitally integrated RFID sensor tags for SHM.

Tag Design	Sensor	Energy Source	Reading Distance	Performance	Ref.
Rocky100 RFID chip and MSP430FR2433	semiconductor strain transducer	RF energy	60 cm. (ImpinJ R420 reader, Seattle, WA, USA)	the relative error of strain monitoring is better than 20.5% (<200 με), 6% (200–450 με) and 2.2% (>450 με)	[82]
SL900A tag	strain gauges	RF energy	n/a	good linearity and a high reliability	[78]
commercial NXP G2iL chip-based tag	strain gauges	RF energy or battery assisted	longer than 20 m	the ability to handle time varying strain	[79,80]
ImpinJ Monza-X chip-based tag	strain gauges and acceleration sensor	RF energy and battery	1.5 m	monitoring of natural frequency of infrastructure	[81]
Monza X-8K RFID unit	ADXL372/ADXL345 accelerometer sensor	solar power/RF energy	up to 17 m	the deep belief network is used to feature extraction; high diagnosis accuracy	[83,84,85,86]
nRF24L01	ADXL345 accelerometer sensor	piezoelectric energy harvester/vibration energy	13 m	reducing the signal dimension through compressed sensing; different algorithms were exploited to improve the performance of fault prediction and diagnosis	[87]
CC430F5137 wireless transceiver	ADXL362 accelerometer	RF energy	2.3 m	up to 500 working cycles per second; energy conversion efficiency of 25%	[77]

**Table 6:** The encoding techniques for chipless RFID.

Encoding Methods	Principle	Pros	Cons
shape-based	the electromagnetic (EM) signature of a specific shape	simple	low coding density
time domain	the duration and interval of the reflected signal	long reading distance; low energy demand	coding capacity and coding density are small; high requirement for readers
frequency domain	encoding data into spectrum using different resonant structures	large storge potentials; high coding density	large spectrum and wideband dedicated reader required
amplitude/phase domain	phase or amplitude modulation of the RCS can be achieved by changing the impedance of the tag antenna	occupy small spectrum resources; simple structure	small coding capacity; additional components required
hybrid	using more than one domain in coding	the data capacity can be greatly increased	complex design

**Table 7:** Chipless RFID sensor tags for SHM.

Tag Design	Sensing Parameters	Sensing Variables	Sensitivity	Application Notes	Ref.
circular patch antenna on RT/duroid 5880 substrate	strain	S-parameters	0°: −1.218 kHz/με; 15°: −1.064 kHz/με; 30°: −0.881 kHz/με; 45°: −0.375 kHz/με; 60°: −0.054 kHz/με; 75°: 0.068 kHz/με; 90°: 0.415 kHz/με	frequency band: 1~3 GHz; read range: 50 mm; strain magnitude and direction detection; different sensitivities in various directions; the existence of rotating parts	[25]
rectangular loop with finger capacitor on flexible polydimethylsiloxane (PDMS) substrate	strain	RCS	n/a	frequency band: 1–1.8 GHz; normal and shear strain detection in all directions; flexible and fully printable; not suitable for metal surfaces	[21]
dual-resonant CMPA on RT/duroid 5880 substrate	strain	RCS	horizontal strain: 528 Hz/με; vertical strain: 384 Hz/με	bits encoded: 4 bits; frequency band: 3–5 GHz; read distance: 3–5 mm; strain magnitude and direction detection; separated layers structures; low sensitivity	[98]
CMPA and tip loaded dipoles on Taconic CER-10-0500 laminate	crack	RCS	horizontal crack: 13.43 MHz/0.1 mm; vertical crack: 6.67 MHz/0.1 mm	bits encoded: 4 bits; frequency band: 2–6 GHz; read distance: 30 cm; crack orientation and width detection; the cracks behind the ID resonators are non-detectable; unreliable for real environment	[99]
CMPA and six inverted “U” and “L” shaped resonators on Rogers RT/duroid 5880	temperature and crack width	RCS	−58.8 MHz/0.1 mm	bits encoded: 7 bits; frequency band: 2–8 GHz; read distance: 65.33 mm; multiple parameters detection; the cracks behind the ID resonators are non-detectable	[92]
frequency selective surface	the increase of corrosion layer thickness	S-parameters	17.6 MHz/month; 1.34 MHz/μm	frequency band: 2–6 GHz; read distance: 150 mm; fused resonance frequency using simple sum; the cross-polarization reading technique; limited reading range	[100]
surface acoustic wave RFID tag with modulator circuit	vibration	time domain signals	n/a	frequency band: 2–3 GHz; modulated by vibration sensor; high vibration rate measurement up to 5 kHz; contact sensor (affect device rotation)	[101,102]

**Table 8:** Pseudocode of the interrogation reader.

For frequency = start 902: step 0.5: end 928MHz.
For power = start 5: step 0.5: end 30dBm.
Query: reader sent request.
If tag respond:
Save the received data.
End if.
End Query.
Next power.
Next frequency.

## Data Availability

Not applicable.
